# Optimization of Ultrasonic-Assisted Extraction, Structural Characterization, and Bioactivities of Polysaccharides from *Nitraria tangutorum* Bobr.

**DOI:** 10.3390/antiox15080986

**Published:** 2026-08-09

**Authors:** Baotang Zhao, Faqin Tao, Shengfang Wang, Wendi Jin

**Affiliations:** 1 College of Food Science and Engineering, Gansu Agricultural University, No. 1 Yingmen Village, Anning District, Lanzhou 730070, China; 2 College of Life Science, Northwest Normal University, Lanzhou 730070, China

**Keywords:** *Nitraria tangutorum* Bobr., polysaccharide, immunomodulation, bioactivities

## Abstract

*Nitraria tangutorum* Bobr. fruits are rich in bioactive polysaccharides (QBC) with high nutritional and developmental potential. This study optimized the ultrasonic-assisted extraction QBC via RSM, determining the optimal parameters to be 45 min, 59 °C, 19:1 mL/g, and 50% (150 W eq.), yielding 15.13%. Structurally, QBC exhibited a Mw of 1.997 × 10^5^, Mw/Mn of 3.727, Rz of 15.6, and a monosaccharide molar ratio of rhamnose:arabinose:mannose:glucose:galactose = 1:1.12:1.66:7.3:1.86. In this study, the effects of QBC on oxidative stress markers and pro-inflammatory cytokines were evaluated via in vivo experiments. The results demonstrated that QBC significantly improved the thymic index and restored immune function. Specifically, QBC upregulated the expression of pro-inflammatory cytokines such as IL-6, TNF-α, and IFN-γ. The analysis of α/β diversity via 16S rRNA sequencing revealed that the CTX group induced a reduction in microbial richness, a more dispersed community structure, and increased intergroup dissimilarities. KEGG functional prediction indicated that polysaccharides regulated the ‘Infectious disease: parasitic’ and ‘Nitrotoluene degradation’ pathways, potentially by exerting their effects through metabolite-mediated immune regulation.

## 1. Introduction

The genus NitrariaL. is classified within the family *Zygophyllaceae* (the caltrop family). This genus consists of perennial deciduous shrubs, and China is home to eight native species of this genus [1,2]. These species represent a crucial component of desert vegetation in China, predominantly distributed across the northwestern and northern regions, including provinces such as Gansu, Xinjiang, and Inner Mongolia [3,4,5]. White-thorn species (*Nitraria* spp.) demonstrate exceptional resilience to multiple abiotic stresses, including drought, high temperatures, extreme cold, saline–alkaline environments, nutrient-deficient soils, and wind–sand erosion. These attributes render them valuable species for ecological engineering applications, particularly in windbreak and sand fixation, as well as saline–alkali land remediation [6,7,8,9,10].

The genus *Nitraria* spp. (commonly referred to as white thorn) contains a range of bioactive components, including sugars, lipids, proteins, retinol, riboflavin, calcium, sodium, and other constituents [11,12,13], and demonstrates significant medicinal properties [14]. It has a long-standing history of medicinal application among ethnic minorities in Northwest China, supported by extensive documentary evidence [15]. It is primarily indicated for treating physical debility, the dual deficiency of qi and blood, spleen–stomach disharmony, dyspepsia, irregular menstruation, and cold-induced lumbago [16,17,18,19]. The fruit of *Nitraria tangutorum* Bobr. has been utilized in folk medicine to address splenic and gastric weakness, indigestion, neurasthenia, and common colds. The leaves are employed as a folk remedy for muscle spasms, neuralgia, and cardiac arrhythmias.

*Nitraria tangutorum* Bobr., a deciduous shrub of the genus Nitraria, is a medicinal and food-source plant with notable ecological, economic, and medicinal significance [7]. It is predominantly distributed in arid deserts, alpine zones, and saline–alkali habitats [7,20]. Polysaccharides extracted from *Nitraria tangutorum* Bobr. constitute one of its primary bioactive constituents, demonstrating substantial potential for disease prevention and treatment, including immunomodulatory, antioxidant [21], anti-inflammatory [22] and hypolipidemic [23]. Polysaccharides are essential biomolecules involved in cellular communication, adhesion, and immune molecular recognition, thereby modulating the immune responses and offering considerable research and application value [2,24]. Higher plant polysaccharides have been utilized as safe alternatives for immunoregulation, antitumor therapy, and trauma treatment. In contrast to bacterial polysaccharides and synthetic immunomodulators, polysaccharides from higher plants exhibit low toxicity and minimal side effects [2,25,26,27,28].

Polysaccharides derived from various traditional medicinal plants exhibit significant immunomodulatory effects in both in vivo and ex vivo settings, demonstrating their potential as therapeutic immunomodulatory agents [29,30]. The existing literature indicates that macrophages play a pivotal role in host defense mechanisms, wherein polysaccharides elicit primary immune responses through the activation of macrophages, B lymphocytes, and other immune cells [31,32]. Upon activation, macrophages secrete a spectrum of cytokines, which subsequently mediate diverse biological effects. Key effector molecules, including nitric oxide (*NO*), tumor necrosis factor-α (*TNF-α*), and reactive oxygen species (ROS), contribute to nonspecific immune defenses by inhibiting tumor growth and combating infections [33,34,35,36,37]. For instance, Astragalus polysaccharide has been shown to function as an immunomodulator in murine peritoneal macrophages, enhancing lysosomal enzyme activity, stimulating the production of *NO*, *ROS*, and *TNF-α* in vitro, and thereby activating nonspecific immune responses. Specifically, Astragalus polysaccharide induces the expression of inducible nitric oxide synthase (*iNOS*), leading to increased *NO* production in macrophages [38,39,40].

Notably, no domestic or international studies have yet reported on the immunomodulatory properties of polysaccharides extracted from *Nitraria tangutorum* Bobr. Based on this, this study optimized the ultrasound-assisted extraction process for QBC and characterized its basic structural features; simultaneously, a cyclophosphamide-induced immunosuppressed mouse model was established to systematically investigate the protective effects of QBC against immune dysfunction and gut microbiota imbalance, as well as its potential mechanisms of action. This study fills a research gap regarding the immunomodulatory functions of Tangut white thorn polysaccharides and provides important theoretical support for their development as natural immunomodulators.

## 2. Experimental Section

### 2.1. Materials

Experimental animals—male mice (SPF grade), 60 mice (22–25 g)—were bought from the Experimental Animal Centre of Gansu Province College of Traditional Chinese Medicine.

QBC: A single fraction of *Nitraria tangutorum* Bobr. polysaccharide obtained by ultrasonic extraction and analyzed and purified on a DEAE-52 chromatography column.

The rest of the materials are as follows: 786-O, MGC803, Hela cells (preserved at the Molecular Biology Laboratory, Institute of Modern Physics, Chinese Academy of Sciences, Lanzhou, Gansu, China), Cyclophosphamide (Sigma-Aldrich, St. Louis, MO, USA), SOD, MDA, MPO, NO, NOS, XOD ELISA kits (Nanjing Jiancheng Bioengineering Institute, Nanjing, Jiangsu, China), IL-6, IL-6, IFN-γ, TNF-α ELISA kits (Beijing Sizhenbai Biotechnology Co., Ltd., Beijing, China). UV-Vis Spectrophotometer (UV1000, LabTech Instruments, Tianjin, China), Constant Temperature Water Bat (HH-S6, Gongyi Yuhua Instrument Factory, Zhengzhou, Henan, China), Multifunctional Enzyme Labeler (Model 550, Bio-Rad Laboratories, Hercules, CA, USA), Recirculating water multi-purpose vacuum pump (SHB-III, Zhengzhou Great Wall Scientific Industry & Trade Co., Zhengzhou, China), ultrasonic processor (QSD-1000, Guangzhou Sciensonic Equipment Co., Ltd., Guangzhou, China), Freeze dryer (LGJ-18S, Hunan Xiangyi Laboratory Instrument Co., Ltd., Changsha, Hunan, China), Freezing centrifuge (DF-101B, Gongyi Yingfeng Instrument Factory, Zhengzhou, China), Thermostatic magnetic stirrer (DF-101B, Gongyi Yuhua Instrument Factory, Zhengzhou, Henan, China).

### 2.2. Methods

#### 2.2.1. Experimental Design and Statistical Analysis

The primary factors influencing polysaccharide extraction yield from *Nitraria tangutorum* Bobr. via ultrasonic-assisted extraction with a maximum rated output of 300 W (100%W eq.) were identified as solid-to-liquid ratio, temperature, time, and ultrasonic power. Based on single-factor experimental results, the experimental design was established using the Box–Behnken central composite design (*BBD*) principle, a widely adopted methodology for optimizing multi-factor processes [41,42,43].

#### 2.2.2. Monosaccharide Composition Analysis

The monosaccharide composition of the polysaccharide fraction (QBC) was determined following protocols detailed in our prior publications [41,42,44].

#### 2.2.3. Fourier Transform Infrared Spectroscopy (FT-IR)

Dried samples were ground with KBr (1:100, *w*/*w*), pressed into pellets, and subjected to *FT-IR* analysis using a Thermo Nicolet iS10 spectrometer (scan range: 400–4000 cm^−1^, 16 scans, 4 cm^−1^ resolution) [41,42,44].

#### 2.2.4. Molecular Weight Determination

Molecular weight was determined using a size-exclusion chromatography–light scattering (SEC-LLS) system, which combines gel permeation chromatography with static light scattering detection for accurate polymer characterization [41,42].

#### 2.2.5. Immunosuppressed Mouse Model Established

Sixty SPF mice were randomly allocated into six groups (*n* = 10 per group) with comparable baseline body weights. All immunosuppressed groups followed an identical CTX exposure schedule differing only in oral treatment. Group 1 (Normal control) received 0.85% sterile saline (20 mL/kg) by oral gavage once daily on Days 1–6, followed by sham intraperitoneal (i.p.) saline injection on Day 6 (2 h after the final gavage); Group 2 (CTX model) received saline gavage (20 mL/kg/day, Days 1–6) plus a single i.p. injection of cyclophosphamide (CTX, 100 mg/kg) on Day 6; Group 3 (MSP positive control) received Lentinula edodes polysaccharide (MSP, 100 mg/kg/day) by gavage (Days 1–6) plus a single i.p. CTX (100 mg/kg) injection on Day 6; Group 4 (CY + QBC-L) received *Nitraria tangutorum* fruit polysaccharide (QBC, 100 mg/kg/day) by gavage (Days 1–6) plus single i.p. CTX (100 mg/kg) on Day 6; Group 5 (CY + QBC-M) was on the same schedule as above with QBC at 200 mg/kg/day; and Group 6 (CY + QBC-H) was on the same schedule as above with QBC at 400 mg/kg/day. All mice were euthanized under anesthesia following orbital blood collection on Day 9 [41,45]. Body weights were recorded, and thymus glands were excised and weighed to calculate the thymus index. The animal experiments for this project were conducted at the animal research facilities of the Lanzhou Veterinary Research Institute, Chinese Academy of Agricultural Sciences. This study was approved by the Animal Ethics Committee of the Lanzhou Veterinary Research Institute, Chinese Academy of Agricultural Sciences (Ethics Review Number: LVRIAEC-2025-157).

#### 2.2.6. Measurement of Enzyme Activity

Referring to the description of Zhuang et al. [46] with adjustments, pre-prepared thymus tissue homogenate supernatants and sera were tested according to the MDA kit instructions. Referring to the means of Liang et al. [47] with minor adjustments, 10% of the tissue homogenate was diluted to 1% of the tissue homogenate with 0.85% saline. *SOD* content was determined according to the instructions of the kit. Thymus tissue samples were pre-treated and subjected to relevant assays according to the MPO assay kit instructions. The supernatant of thymus tissue prepared was taken and the relevant levels were measured using *NO* and *iNOS* kits, respectively. Reference can be made to Wang et al. and Yang et al. [48,49].

#### 2.2.7. Measurement of Cytokine IL-6, TNF-α and IFN-γ Content

The supernatants of thymus tissues prepared were selected for the determination of *IL-6*, *TNF-α* and *IFN-γ* using the *IL-6*, *TNF-α* and *IFN-γ* kits, respectively, according to the requirements of the different kits.

#### 2.2.8. Determination of Thymus Histopathology in Mice

In accordance with the methodology previously reported by this laboratory [50], histological sections of the thymus were processed, stained with hematoxylin and eosin, and examined under a microscope. Specifically, fresh thymus tissue was washed with pre-chilled saline and then placed in a 4% paraformaldehyde fixative for 24–48 h. The tissue was dehydrated with ethanol and cleared with xylene. The cleared tissue blocks were then immersed in molten paraffin for infiltration and embedding. After sectioning, the sections were dried; following dewaxing, they were stained with hematoxylin and eosin. Finally, the sections were re-dehydrated and cleared, mounted with neutral resin, and examined under a microscope.

#### 2.2.9. 16S rRNA Gene Sequencing Analysis of Gut Microbiota

To investigate the polysaccharide-induced modulation of gut microbiota, three key experimental groups from the mouse trial were selected for detailed 16S rRNA gene sequencing analysis: NC group (Normal control): mice maintained under standard conditions without pharmacological intervention, representing baseline gut microbiota composition; *CTX* group (Cyclophosphamide-induced model): immunosuppressed mice receiving cyclophosphamide to induce dysbiosis, modeling microbiota disruption; and QBC group (Polysaccharide treatment): mice administered with QBC (200 mg/kg/day) + *CTX* (100 mg/kg/day) via gavage on days 1–6 to assess microbial restorative effects. Total microbial DNA was extracted from cecal contents using the Mag-bind Soil DNA Kit (Omega Bio-tek, Norcross, GA, USA) combined with the Shi and Qiu’ method [51,52]. Genomic DNA was extracted from ~200 mg frozen fecal pellets (*n* = 10 biologically independent mice per group, 30 samples total) using the Mag-Bind Soil DNA Kit (Omega Bio-tek, Inc., Norcross, GA, USA), and the V3–V4 hypervariable region of the bacterial 16S rRNA gene was amplified with barcoded primers 338F/806R under standard cycling conditions, with negative no-template controls included in all batches to monitor contamination. Purified amplicons were used to construct libraries with the TruSeq Nano DNA LT Library Prep Kit, and paired-end 2 × 250 bp sequencing was performed on an Illumina NovaSeq 6000 platform (Illumina, Inc., San Diego, CA, USA). Raw reads were processed via QIIME 2 (v2023.5) and denoised using DADA2 (v1.28.0) to generate Amplicon Sequence Variants (ASVs) at single-nucleotide resolution, retaining 742,158 high-quality reads with an average of 24,739 reads per sample (range: 21,458–28,112); the ASV table was rarefied to the minimum sequencing depth (21,458 reads) prior to analysis, and taxonomic annotation was performed against the SILVA rRNA database (release 138) with a 0.7 confidence cutoff. Alpha diversity differences across groups were assessed via the Kruskal–Wallis test with Dunn post hoc test and Benjamini–Hochberg false discovery rate (FDR) correction; beta diversity was evaluated using Bray–Curtis dissimilarity with PERMANOVA (999 permutations) and ANOSIM, both FDR-corrected. Differential abundant taxa were identified using LEfSe (LDA score threshold = 2.0) and STAMP (with Wilcoxon rank-sum or Kruskal–Wallis tests plus FDR correction for two- or three-group comparisons, respectively). Potential metabolic functions were predicted using PICRUSt2 (v2.5.0) based on Greengene 13.5, with the explicit caveat that these represent inferred rather than directly measured microbial activities, presented solely as exploratory hypotheses for future validation.

#### 2.2.10. Data Analysis

All experimental data are presented as mean ± standard deviation (mean ± SD). Preliminary data organization was performed using Microsoft Excel 2026; tests for normality, homogeneity of variances, one-way *ANOVA*, and post hoc multiple comparisons were conducted using IBM SPSS Statistics for Windows, Version 26.0 (IBM Corp., Armonk, NY, USA). We first used the Shapiro–Wilk test to assess the normality of the data and the Levene test to evaluate homogeneity of variances. When the data were normally distributed and variances were homogeneous, we used Tukey’s method for post-hoc multiple comparisons between groups. The significance level was set at α = 0.05. Differences were considered statistically significant at *p* < 0.05 (*), *p* < 0.01 (**), and highly significant at *p* < 0.001 (***). Experimental graphs were plotted using *Origin* 2024 software.

## 3. Results

### 3.1. Results of Single Factor Experiments

#### 3.1.1. Effect of Ultrasonication Time on Polysaccharide Extraction Yield

**Figure 1:**
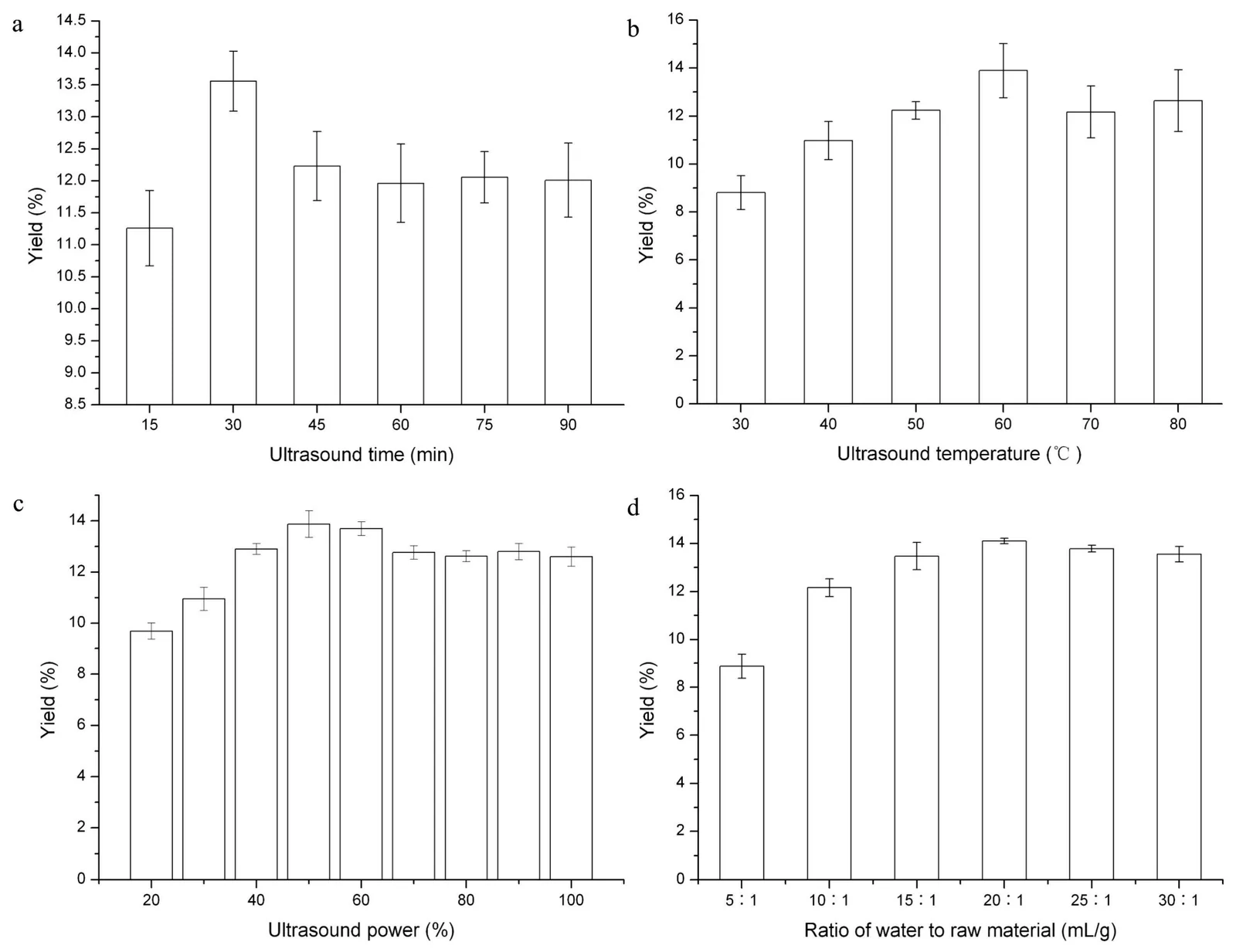
Effects of ultrasonication time (**a**), temperature (**b**), power (**c**), and the water-to-raw material ratio (**d**) on the polysaccharide extraction yield from *Nitraria tangutorum* Bobr.

As shown in Figure 1a, when the ultrasonication time ranged from 15 to 30 min, the yield increased. Subsequently, the yield exhibited a downward trend between 30 and 90 min. This pattern suggests that the polysaccharides were predominantly extracted within the initial 30 min. Considering energy conservation, the need to shorten the production cycle, and the balance between the overall yield, an ultrasonication time of 15–45 min is recommended as the optimal parameter.

#### 3.1.2. Effect of Ultrasonic Temperature on Polysaccharide Extraction Yield

As shown in Figure 1b, the extraction yield increased with rising temperature up to 60 °C. Beyond 80 °C, the yield plateaued without significant fluctuations. Additionally, visual observation of the obtained polysaccharide solutions indicated that the color of the polysaccharides darkened as the temperature increased. Moreover, polysaccharide solutions prepared at lower temperatures were less filterable compared to those at higher temperatures. Considering that elevated temperatures may potentially alter the structural integrity and bioactivity of polysaccharides, a temperature range of 50–70 °C was determined as the optimal extraction temperature.

#### 3.1.3. Effect of Ultrasonic Power on Polysaccharide Extraction Yield

As shown in Figure 1c, the extraction yield decreased with the increase in ultrasonic power. No significant variations in yield were observed at power levels of 50% and 60%. A larger yield of polysaccharides occurred with the stronger extraction power at the early period. However, excessively high extraction power can damage the molecular structure and biological activity of polysaccharides; therefore, a power range of 40–60% is considered the optimal condition.

#### 3.1.4. Effect of Material to Liquid Ratio on Polysaccharide Extraction Yield

As shown in Figure 1d, the extraction yield increases as the solid-to-liquid ratio rises (5:1–20:1). This can be attributed to the increased solubility of polysaccharides at higher moisture contents, which reduces extraction losses. The polysaccharide yield shows a downward trend in the range of 20:1–30:1, which may be due to a weakening of the ultrasonic cavitation effect. After comprehensive consideration, the solid-to-liquid ratio was controlled between 15:1 and 25:1.

### 3.2. Results of the Response Surface Design Experiment

#### 3.2.1. Response Surface Design and Results

Based on the Box–Behnken central composite design principle and the results of single factor experiments, four factors—extraction time (*X*_1_), extraction temperature (*X*_2_), solid–liquid ratio (*X*_3_), and ultrasonic power (*X*_4_)—were selected as significantly influencing the polysaccharide extraction rate of *Nitraria tangutorum* Bobr. Building on the single factor experiments, a four-factor, three-level response surface methodology was adopted. The response surface experimental design and results are provided in Table 1.

#### 3.2.2. Establishment and Analysis of Regression Models

The data in Table 1 were analyzed using SAS statistical software for Windows, Version 9.4 (SAS Institute Inc., Cary, NC, USA) to obtain the analysis of variance (ANOVA) results for the regression model and the quadratic polynomial regression model. The goodness of fit of the regression equation was validated using the determination coefficient (R^2^ 0.9657) and adjusted determination coefficient (R_adj_^2^ = 0.9314) (Table 2). SAS software, a widely recognized statistical tool, was used for these analyses.

*Y* = 14.42 + 0.15*X*_1_ − 0.090*X*_2_ − 0.036*X*_3_ + 0.039*X*_4_ − 0.035*X*_1_*X*_2_ − 0.32*X*_1_*X*_3_ + 0.23*X*_1_*X*_4_ + 0.77*X*_2_*X*_3_ + 0.21*X*_2_*X*_4_ + 0.40*X*_3_X_4_ − 0.44*X*_1_^2^ − 1.15*X*_2_^2^ − 0.86*X*_3_^2^ − 0.70*X*_4_^2^

The extraction yield of *Nitraria tangutorum* Bobr. polysaccharides was set as the response variable, and the regression model was established using the SAS RSREG procedure. The ANOVA results (Table 2) showed that the overall model was highly significant (*p* < 0.0001). The linear terms (*X*_1_), quadratic terms (*X*_1_^2^, *X*_2_^2^, *X*_3_^2^, *X*_4_^2^), and interaction terms (*X*_1_*X*_3_, *X*_1_*X*_4_, *X*_2_*X*_3_, *X*_3_*X*_4_) significantly affected the extraction yield. The high coefficient of determination (R^2^ = 0.9657) demonstrated that 96.57% of the yield variability could be explained by the selected variables, verifying the good fitting ability and reliability of the established regression model.

The theoretically optimal extraction parameters were 45 min, 58.79 °C, a solid–liquid ratio of 18.85:1, and ultrasonic power of 51.11%. For practical experimental operation, the parameters were rounded and adjusted to 45 min, 59 °C, 19:1, and 50% (150 W eq.). Under the modified optimal conditions, the predicted polysaccharide extraction yield reached 15.13%.

**Figure 2:**
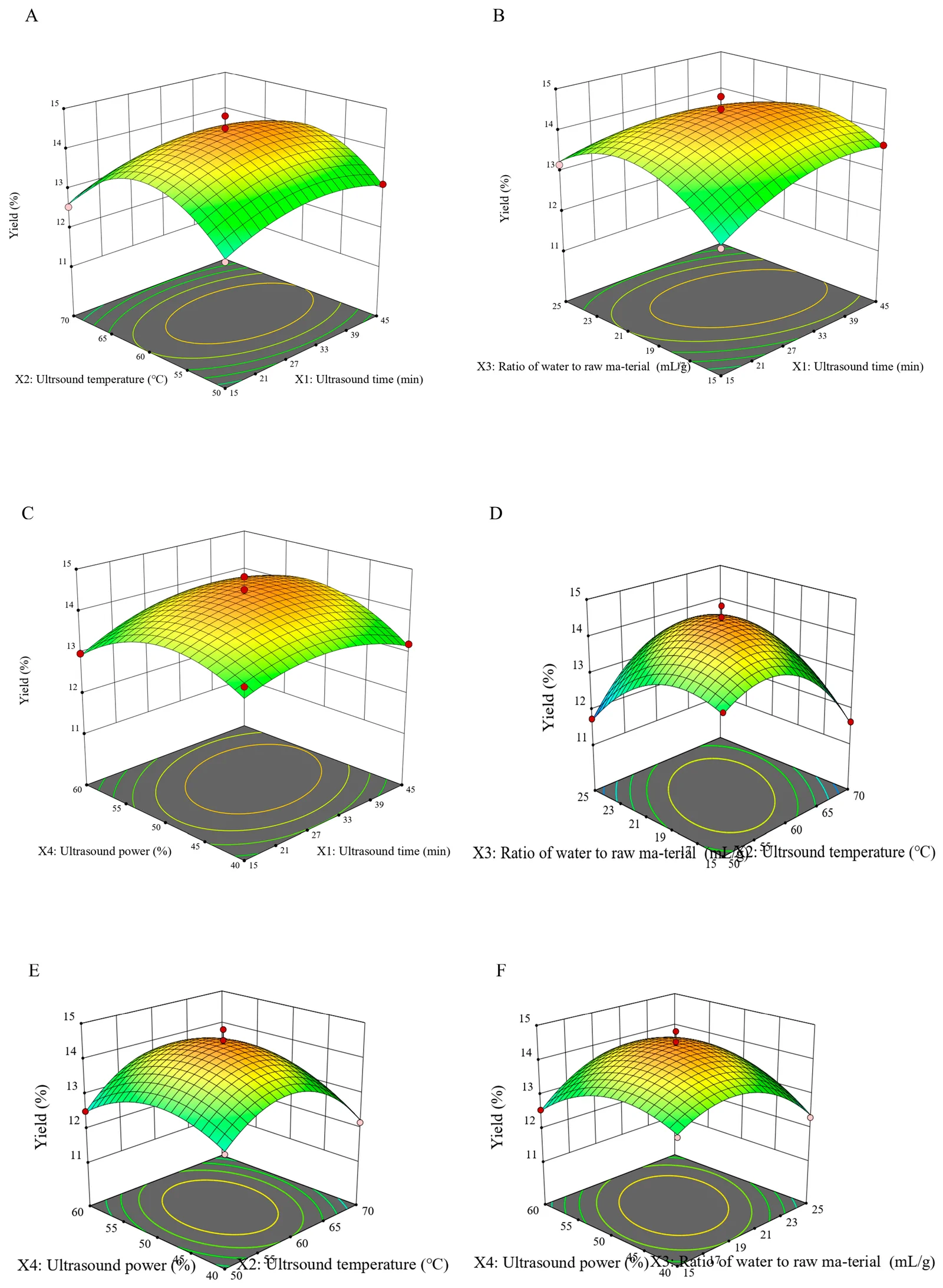
Three-dimensional response surface showing pairwise interactions of extraction variables (*X*_1_–*X*_4_) on *Nitraria tangutorum* polysaccharide yield: (**A**) ultrasound time-ultrasound temperature; (**B**) ultrasound time-ratio of water to raw material; (**C**) ultrasound time -ultrasound power; (**D**) ultrasound temperature-ultrasound power; (**E**) ultrasound temperature-ratio of water to raw material; and (**F**) ratio of water to raw material-ultrasound power.

Figure 2 visually illustrates the effects of various factors on the yield of water-soluble polysaccharide extraction. Figure 2A presents a fully convex parabolic surface. The slope along the ultrasonic temperature (*X*_2_) axis appears visually steeper than along the time (*X*_1_) axis, with a sharper color transition from yellow to red. However, consistent with the ANOVA (Table 2), the linear effect of *X*_2_ was not statistically significant (*p* = 0.1532) and the *X*_1_*X*_2_ interaction term was far from significance (*p* = 0.7380). Thus, the apparent steepness reflects the unmodelled quadratic curvature of the fitted polynomial rather than a true interactive synergy; the ultrasonic temperature showed only a numerical trend towards influencing yield within the tested range. In Figure 2B, the overall surface representing the relationship between the solid-to-liquid ratio (*X*_3_) and *X*_1_ is convex. Although the color variation along the *X*_1_*–X*_3_ plane is not pronounced, the surface has a steeper slope along the *X*_1_ axis and a relatively gentler slope along the *X*_3_ axis, indicating that the ultrasonication time has a greater influence on the yield than the solid-to-liquid ratio, and that there is a significant interaction between the two. Figure 2C clearly shows the surface curves for ultrasonic power (*X*_4_) and *X*_1_. The surface for the *X*_1_ dimension is slightly steeper than that for *X*_4_*,* and the color variation between *X*_1_ and *X*_4_ is not pronounced. The ultrasonic time exerts a stronger regulatory effect on polysaccharide extraction yield; the two-factor surface exhibits significant fluctuations, and the interaction effect is prominent. In Figure 2D, compared to the *X*_3_, the surface along the *X*_2_ axis is steeper, and the color transition from yellow to red is more pronounced. This indicates that the ultrasonic temperature has a significantly stronger effect on the extraction yield than the solid-to-liquid ratio. Furthermore, yields drop sharply in both the low-temperature, low-solid-to-liquid-ratio region and the high-temperature, high-solid-to-liquid-ratio region, demonstrating a clear nonlinear interaction between the two factors. Figure 2E shows a rapid drop-off of the contour ridge along the temperature axis (*X*_2_) relative to ultrasonic power (*X*_4_). While the visual gradient suggests temperature dominates the modelled response, the ANOVA confirms no statistically significant interaction between *X*_2_ and *X*_4_ (*p* = 0.0642, marginal at best). The sharp descent away from the central plateau is attributable to the strong negative quadratic term of *X*_2_ (optimum-centered parabola), not a synergistic coupling of temperature and power. Both factors exert predominantly independent, saturable effects on polysaccharide liberation. Figure 2F shows that the surface in the *X*_4_ dimension is steeper, while the slope in the *X*_3_ dimension is gentler and the color changes are more gradual. This indicates that ultrasonic power has a greater effect on polysaccharide extraction efficiency than the solid-to-liquid ratio. At low power and low solid-to-liquid ratios, polysaccharide elution is insufficient, while excessively high power or solid-to-liquid ratios can easily lead to polysaccharide degradation; there is a significant interaction between the two.

### 3.3. Structural Characterization of QBC

#### 3.3.1. IR Spectra of QBC

**Figure 3:**
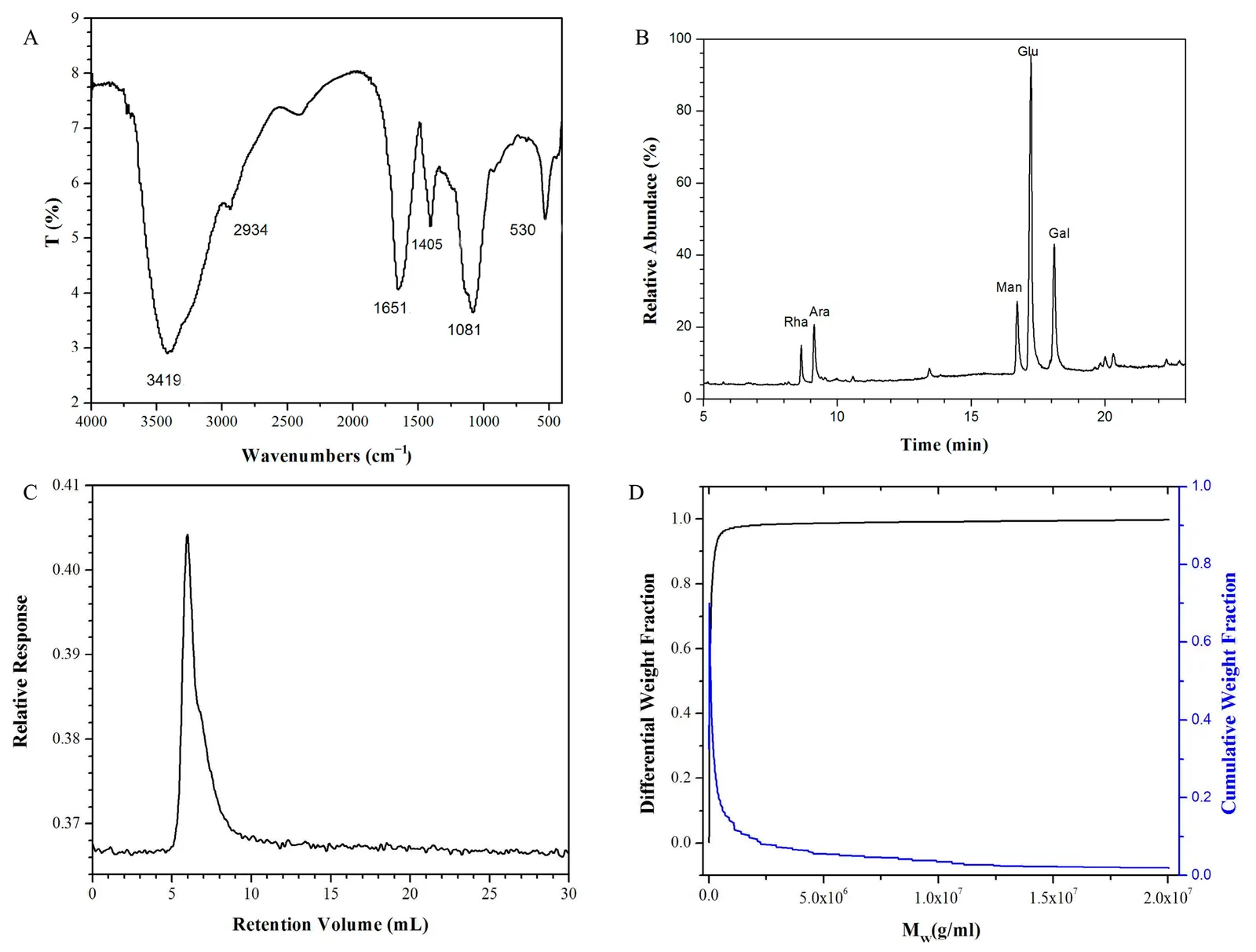
FT−IR spectroscopy (**A**), GC−MS chromatograms of component monosaccharides (**B**), and SEC-LLS chromatograms of QBC (**C**,**D**).

Figure 3A shows the characteristic absorption peaks of carbohydrates. The peaks at 3419 cm^−1^ correspond to the −OH stretching vibration, 1081 cm^−1^ to the furanose stretching vibration, and 2930 cm^−1^ to the C−H stretching vibration. The results indicate that QBC exhibits the characteristic absorption peaks of polysaccharides.

#### 3.3.2. Monosaccharide Profile and Molar Ratio of QBC Fractions

The GC−MS analysis results of QBC after acid hydrolysis and derivatization are shown in the figure. As shown in Figure 3B, QBC consists of five monosaccharides, rhamnose, arabinose, mannose, glucose, and galactose, with a molar ratio of 1:1.12:1.66:7.3:1.86.

#### 3.3.3. SEC−LLS Analysis

Figure 3C shows the SEC-LLS chromatogram of QBC. As shown in the figure, QBC exhibits a single peak, indicating that it is non-aggregated. The weight-average molecular weight (*Mw*) of QBC is 1.997 × 10^5^, the polydispersity (*Mw*/*Mn*) is 3.727, and the gyration radius (Rz) is 15.6.

### 3.4. Effect of QBC on Thymus and Serum SOD Activity in Mice

**Figure 4:**
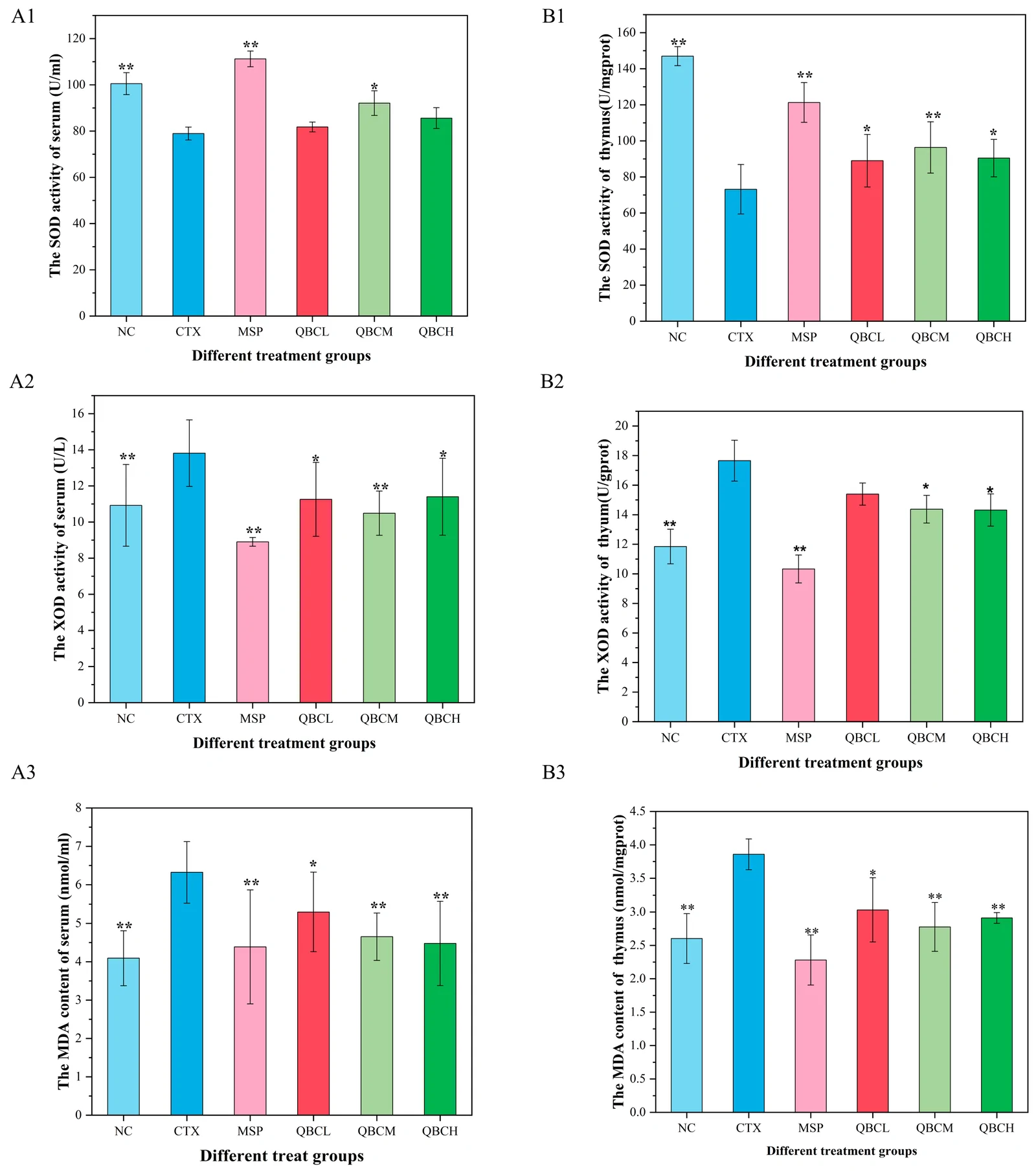
The affections of QBC on *SOD*, *XOD*, and *MDA* content for thymus (**A1**–**A3**) and serum (**B1**–**B3**) of mice. (*p* < 0.05 (*), *p* < 0.01 (**); see methods for criteria).

Often considered indicators of the body’s antioxidant defense system, *SOD* eliminates oxygen free radicals [53]. Figure 4A1 displays the effect of QBC on *SOD* activity in mouse thymus. Compared to the CTX group, the *SOD* activities in the thymus of mice given QBC and MSP alone by gavage were increased to different degrees. Different doses of QBC with *CTX* given to mice by gavage dramatically increased the *SOD* activity in the thymus of mice, with a significant difference in the QBCM group. Figure 4B1 shows the effect of QBC on *SOD* activity in the serum of mice. *SOD* activity in the serum of mice in the control group of MSP, and the three dose groups of QBC, namely, low, medium, and high doses, did not have any noticeable difference compared to that of the normal control group (NC), but there was a significant difference between it and that of the model group (CTX), which indicated that the mouse *SOD* activity in serum was significantly reduced by CTX, but the *SOD* activity in serum of the normal group of mice was not significantly changed by the administration of gavage of natural polysaccharides; therefore, QBC did not affect the activity of *SOD* in serum of normal mice. In the immunosuppressed mice caused by *CTX*, the serum *SOD* activity was slightly increased by either gavage of MSP or QBC; however, the difference was not evident when compared with that of the *CTX* group of mice. The results showed that the QBC fraction isolated from *Nitraria tangutorum* Bobr. had no significant effect on serum *SOD* activity.

### 3.5. Effect of QBC on Tissue and Serum XOD Activity in Mice

Xanthine oxidase (*XOD*) is a class of aerobic dehydrogenases whose activity increases when hepatocytes are damaged [54,55]. As shown in Figure 4A2, *XOD* activity was significantly increased in the thymus tissue of the model group compared to the blank control group. The thymic *XOD* activity of mice treated with QBC alone was markedly lower than that of the *CTX* model group, with statistically significant intergroup differences. The *XOD* activity in the thymus of mice injected with *CTX* and QBC decreased slightly with the gradual increase of the administered dose. The results showed that QBC was able to remove *XOD*, thus lessening the detriment to the thymus caused by superoxide radicals produced by *XOD*-catalyzed xanthine and ensuring the integrity of cell membrane structure and function.

Figure 4B2 shows the effect of QBC on the *XOD* activity in serum of mice. The serum of model group mice was sensibly elevated. This suggests that the model group mice had damage to the thymus due to immunosuppression, resulting in the release of *XOD* into the serum. Compared to the mice in the *CTX* group, the serum *XOD* activity was significantly lower in the NC and polysaccharide control mice, while, for the QBCL group, the serum *XOD* activity was slightly reduced, but the divergence was not prominent. The elevation of *XOD* activity in the serum of immunosuppressed mice was probably due to thymus damage, and, therefore, QBC did not have a significant repairing effect on thymus damage.

### 3.6. Effect of QBC on MDA Content in Mouse Thymus and Serum

*CTX* causes thymus damage, reduces antioxidant enzyme activity, and generates ROS, which causes oxidative stress and increased *MDA* levels [56,57]. The *MDA* levels reflect the degree of free-radical-induced damage [58]. As can be seen from Figure 4A3, under the influence of *CTX*, the *MDA* content in the thymus of the model group mice was dramatically higher than that of the remaining groups of mice, whereas there was no significant change in the mice in the MSP group and the three dosage groups of QBC compared with the NC group. For the immunosuppressed mice given QBC gavage versus the immunosuppressed mice given MSP gavage, the *MDA* content in the thymus was dramatically decreased, and the middle dose group of QBC even reached the normal level. This is likely due to the ability of QBC to simultaneously inhibit lipid peroxidation and scavenge lipid peroxidation products. The *MDA* content in serum of mice in the model group was sensibly higher. This may be due to the enhanced lipid peroxidation capacity in *CTX* -immunosuppressed mice, leading to an increased *MDA* production and, consequently, an increase in the amount of *MDA* entering the bloodstream. Therefore, compared with mice not subjected to *CTX* immunosuppression, their serum MDA levels were significantly elevated. According to the Figure 4B3, the *MDA* content in the serum of mice in the gavage group of MSP and QBC was not significantly different from that of NC, suggesting that QBC does not damage tissues such as the thymus of normal mice and does not trigger excessive lipid peroxidation; the two polysaccharides were comparable in inhibiting lipid peroxidation when compared with the *CTX* group. The difference in the serum *MDA* content of mice in the low-dose QBCL administration group was not significant compared to the *CTX* group, which indicated that the low-dose QBC did not have a remarkable inhibition effect on lipid peroxidation in the serum of mice, and was not able to remove *MDA* from the serum significantly.

### 3.7. Effect of QBC on Thymic Index in Mice

**Figure 5:**
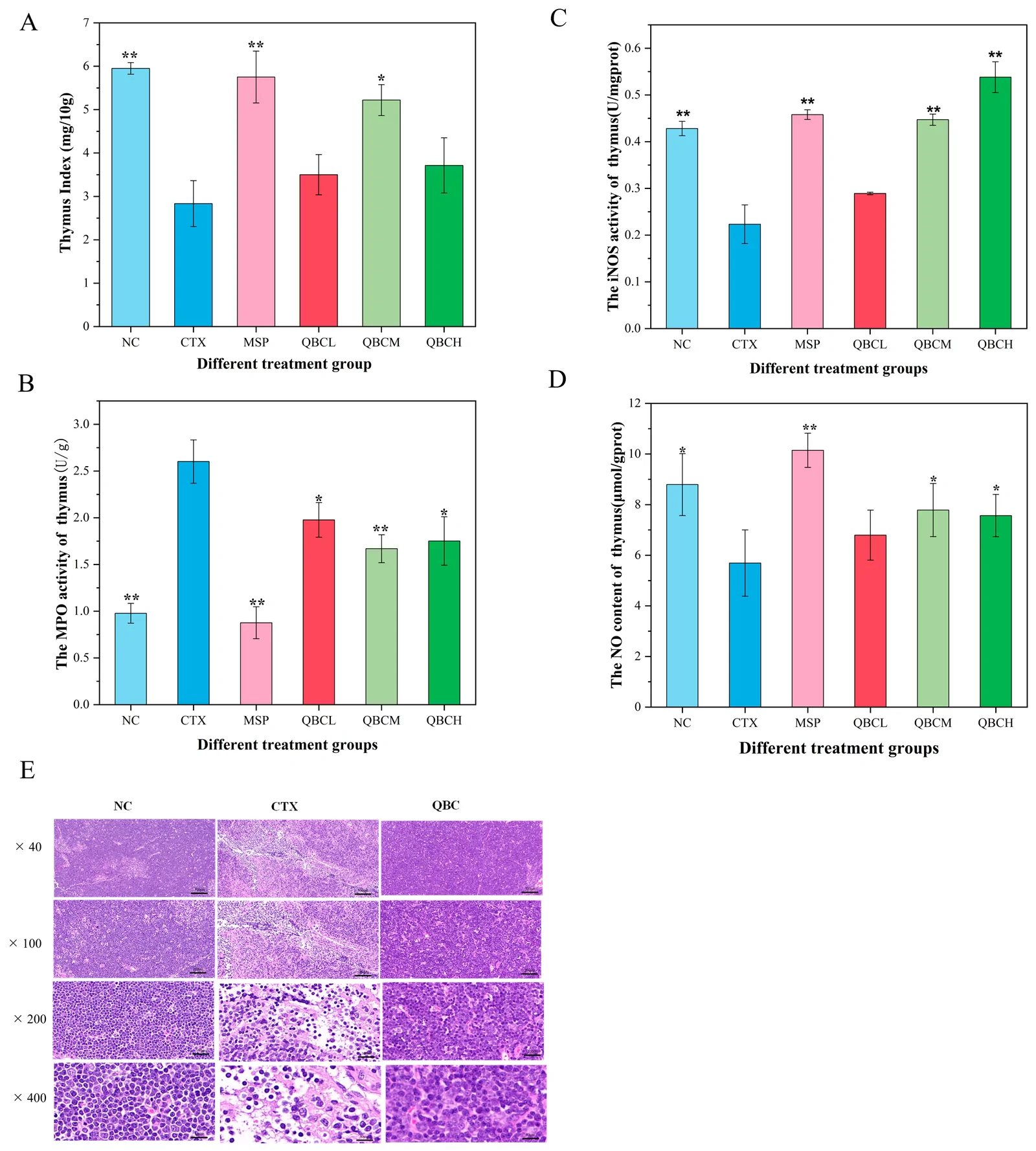
The affection of QBC on thymus index (**A**), *MPO* activity (**B**), *iNOS* (**C**) activity and *NO* content (**D**), and pathological analysis of thymus tissue (**E**). (*p* < 0.05 (*), *p* < 0.01 (**); see methods for criteria).

The thymic index is an essential indicator for assessing the immune function of the body and is extensively used to detect the immune condition of the body [45]. Cyclophosphamide (*CTX*), however, as an immunosuppressant, causes severe damage to the immune and hematopoietic systems, resulting in a decrease in thymic index [53]. As can be seen in Figure 5A, the difference in the thymic index between the model group mice and the blank group mice was extremely significant, indicating that *CTX* successfully established an immune injury model in mice. The thymic indices of mice in the blank control group, the MSP control group and the three doses of QBC group were higher than those of the mice in the model group, and the distinction was highly notable, whereas the thymic indices of the MSP group injected with *CTX* and the QBC administration group were elevated, with the exception of the QBCM group; the difference was not significant. This indicates that the thymus, although recovered after immune injury, could not be immediately restored to the normal level. Therefore, both MSP and QBC were slow to repair the thymus.

### 3.8. Effect of QBC on MPO Activity in Mouse Thymus

MPO is mainly found in immune cells such as neutrophils and monocytes. Changes in MPO activity in the thymus can reflect, to some extent, the degree of activation of immune cells and the strength of the immune response [59,60]. As can be seen in Figure 5B, MPO activity in the thymus of mice after immunosuppression with *CTX* was significantly elevated, with a highly huge discrepancy between the blank control group and the polysaccharide administration group alone; whereas there was no noteworthy difference in the MPO level between the administration group alone and the normal group. MPO activity was significantly reduced after administration of QBC to mice after immunosuppression for *CTX*, but remained above normal levels. The results suggest that polysaccharides do not cause disturbances in MPO activity in the thymus of normal mice, but have a remarkable inhibition effect on MPO activity in the thymus of immunosuppressed mice.

### 3.9. Effect of QBC on iNOS and NO Content in Mouse Thymus

Nitric oxide (*NO*), also known as vascular endothelial diastolic factor, is produced mainly in vascular endothelial cells, smooth muscle cells, etc. It is a highly reactive free radical present in living organisms and plays a very important role in immune signaling, as well as acting as a second messenger with neurotransmitter properties [61,62]. As an effector molecule, *NO* can inhibit platelet aggregation, and mediate cytotoxic effects and immunomodulation [63,64]. *NO* can initiate different signaling pathways through the mitogen-activated protein kinase pathway (MAPK), altering the corresponding functions of the organism for signal regulation, as well as mediating the regulation of cellular functions by elevating the level of intercellular cGMP, mediating phosphodiesterase and other pathways. NO production, however, is influenced by the activity of inducible nitric oxide synthase (*iNOS*) in macrophages in addition to the substrate molecule L-Arg [65,66].

As shown in Figure 5C, the content of *NO* in the thymus of *CTX mice* decreased significantly, whereas the *NO* content in the thymus of mice in the administered group tended to be at the NC level. This is likely because the use of *CTX* can kill immune cells, and the thymus belongs to the immune organs, which will also be damaged by *CTX*, leading to a decrease in the number of immune cells, for instance macrophages and T cells, which, in turn, will lead to a decrease in the amount of *NO* produced by the immune cells, and a decrease in the immune regulatory function mediated by the *NO* molecule, resulting in suppression of the immune function of the mice. The injection of distinct doses of QBC into mice with immune injury caused by *CTX* was able to stimulate the secretion of the second messenger *NO* by immune cells. As a result, the *NO* content in the thymus of the rat was little by little recovered to the normal level. The *NO* content in the thymus of mice injected with dissimilar dosages of QBC alone was not significantly elevated compared with that of the NG group, and, thus, did not result in excessive *NO* that could lead to the advancement of disease.

As can be seen in Figure 5D, the viability of *iNOS* in the thymus of six groups of mice, NC, MSP, QBCM, and QBCH, showed highly significant differences compared with CTX. *INOS* viability was close to each other between the three groups, QBCM and QBCH, and the differences were significant compared with CTX. The results suggest that QBC stimulates the production of *INOS* in the mouse thymus, which, in turn, produces the signaling molecule NO for immunomodulation.

### 3.10. Effects of QBC Gastric Administration on Pathological Changes in Thymus

The HE staining of rat thymus tissue is shown in Figure 5E. In a normal thymus, densely packed thymic epithelial cells (TECs) form a reticular network enveloping the immature T cell precursor. The thymus exhibits full volume with a distinct demarcation between the cortex and medulla. TECs are tightly arranged, forming a complete reticular scaffold. The medulla contains numerous CD4^+^/CD8^+^ T cells, and thymic corpuscles are intact. T-cell-negative and -positive selection proceeds normally, indicating robust immune responsiveness. In *CTX*-induced mice, thymic cortex atrophy, reduced TECs, a disrupted reticular structure, a loss of immature T cells, and a hollow medulla are observed. Mature T cells are significantly depleted, macrophage infiltration increases, thymic corpuscles disappear, and structural disintegration occurs. Eosin staining reveals widened intercellular spaces, impaired T-cell development, extremely low immune response capacity, and susceptibility to infection. In the QBC-treated group, thymic cortex partially recovered, the reticular structures were rebuilt, the medulla regenerated, a small number of new thymic lobules formed, macrophage activity increased (with enhanced phagocytic granules), interstitial inflammation subsided, edema resolved, and neutrophil infiltration decreased. This indicates a restored T-cell proliferation capacity, elevated immunoglobulin (e.g., IgG) levels, and reduced infection risk. Hematoxylin and eosin (HE) staining revealed pathological alterations in *CTX*-induced immunodeficient mouse thymuses, including cortical atrophy, medullary emptying, and the disappearance of thymic lobules. Polysaccharide healing significantly ameliorated the thymic structure and function through antioxidant and anti-inflammatory effects, the promotion of cellular regeneration, and microenvironment remodeling. The mechanism involves the activation of the Nrf2 pathway, inhibition of the NF-κB pathway, and regulation of immune cell signaling pathways, offering new strategies for immunodeficiency and tumor immunotherapy.

### 3.11. Effect of QBC on Tissue IL-6, TNF-α, and IFN-γ Content in Mice

Interleukin-6 (*IL-6*) can promote B-cell differentiation and antibody secretion, and enhance T-cell activation and proliferation; tumor necrosis factor-α (*TNF-α*) can activate endothelial cells and promote inflammatory cell adhesion and migration; and Interferon-γ (*IFN-γ*) can activate macrophages, enhance their ability to phagocytose and kill pathogens, and facilitate the differentiation of Th1 cells [67,68,69].

As can be seen from Table 3, the levels of *IL-6*, *TNF-α*, and *IFN-γ* in the thymus of mice in the MG group were reduced as a result of immune injury by CY compared to mice in the NC group. The levels of *TNF-α* and *IFN-γ* in the thymus of mice administered alone did not change significantly compared with the NC group, whereas the levels of *IL-6* were significantly higher. The levels of *IL-6* in the thymus of mice immunocompromised by *CTX* were, little by little, recovered to natural levels by QBC, and the levels of IFN-α gradually approached those of the NC group. The results showed that QBC did not cause the excessive production of *TNF-α* in the thymus and thymus to produce inflammatory exudation in the organism, and also likely did not stimulate neutrophils to secrete excessive myeloperoxidase. However, in mice with immunosuppression caused by *CTX*, *TNF-α* promoted the differentiation of myeloid cells to macrophages and enhanced the immune function of the mice. *IFN-γ* and *IL-6* were sufficient to synergistically induce QBC and promote the production of antibodies by the immune cells or the killing of pathogenic microorganisms, improving the immune function.

### 3.12. Effect of QBC on Gut Microbiota

**Figure 6:**
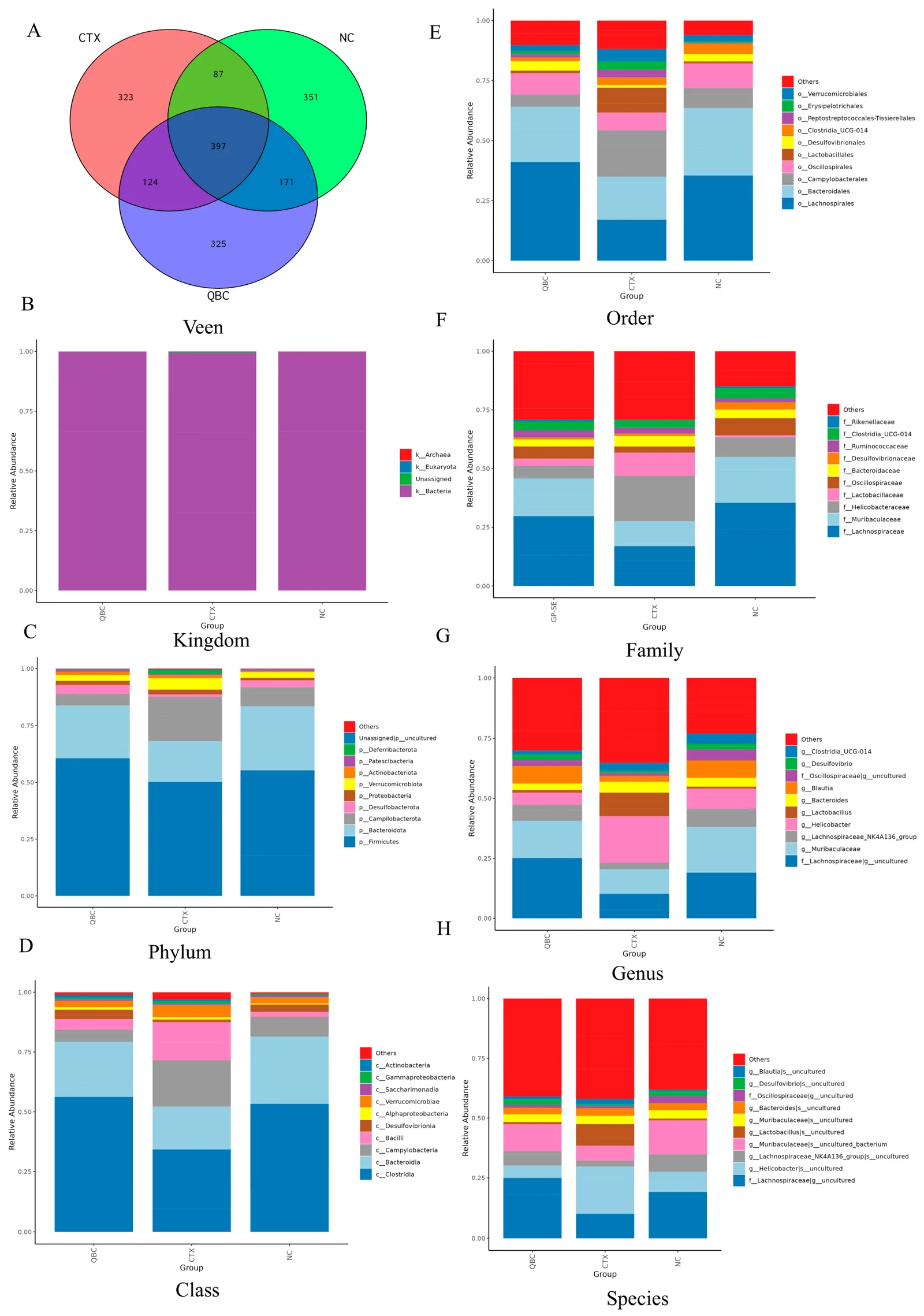
Analysis of intestinal microbial diversity and species composition: (**A**) Venn diagrams of OTUs, and (**B**–**H**) microbiota compositions at the Kingdom, Phylum, Class, Order, Family, Genus, and Species level.

The Venn diagram (Figure 6A) analysis reveals that a total of 1778 operational taxonomic units (OTUs) were identified across the three experimental groups (NC, CTX, and QBC). The specific OTU distributions are as follows: the NC group contained 1006 OTUs, the *CTX* group contained 931 OTUs, and the QBC group contained 1017 OTUs. Meanwhile, the overlap between NC and *CTX* groups comprised 484 OTUs, the NC and QBC groups shared 568 OTUs, and the overlap between the QBC and *CTX* groups comprised 521 OTUs. The core OTUs shared by all three groups amounted to 397. The results indicate that administration of the immunosuppressant (*CTX* group) led to a reduction in gut microbial diversity, as evidenced by the lower OTU count in the *CTX* group (931 OTUs) compared to the NC group (1006 OTUs). In contrast, after therapeutic intervention (QBC group), the OTU count increased to 1017, suggesting a partial restoration of microbial richness. This pattern likely demonstrates that the treatment mitigated the immunosuppressant-induced dysbiosis, promoting the recovery of the gut microbiota composition. At the kingdom level (Figure 6B), the Bacteria phylum accounted for 99.96%, 99.39%, and 99.94% of the total in the NC, CTX, and QBC groups, respectively, indicating that bacteria constitute the predominant component of the gut microbiota in these three groups. From a statistical analysis of the top 10 bacterial phyla (Figure 6C), it is evident that they account for 91.77%, 87.52%, and 88.78%, respectively. Among these, the dominant phyla Firmicutes, Bacteroidota, and Campylobacterota in the NC, CTX, and QBC groups were (55.30%, 28.14%, 8.33%), (50.17%, 18.00%, 19.35%), and (60.64%, 23.09%, 5.06%), respectively.

It is evident that the proportion of *Bacteroidota* in the *CTX* group decreased, while the two dominant bacterial groups rebounded after treatment with polysaccharide. At the class level (Figure 6D), the top 10 bacterial groups were analyzed. Among these, the proportion of *Clostridia, Bacteroidia,* and *Campylobacteria* accounted for 89.75%, 71.66%, and 84.33% of the total, respectively, in the NC, CTX, and QBC groups. In the intestines of mice with CTX-induced colitis, the proportion of Bacilli increased from 2.02% in the NC group to 15.94%, and decreased to 5.05% after *CTX* treatment. In the gut microbiome, *Firmicutes, Bacteroidota,* and *Campylobacterota* are the three core phyla; their abundance, metabolic functions, and interactions with the host directly influence immune homeostasis. At the order level (Figure 6E), the proportions of bacteria belonging to the four orders (*Lachnospirales*, *Bacteroidales, Campylobacterales* and *Oscillospirales*) were 81.13%, 61.68%, and 78.04% in the NC, CTX, and QBC groups, respectively. Specifically, there were no significant differences in the proportions of these four bacterial orders (*Lachnospirales, Bacteroidales, Campylobacterales*, and *Oscillospirales*) between the NC group and the QBC group, but, in the *CTX* group, *Lachnospirales* (16.97%) and *Bacteroidales* (17.99%) showed a significant decrease, while *Campylobacterales* (19.35%) showed a significant increase. At the family level (Figure 6F), bacteria belonging to the 10 families accounted for 85.05%, 70.87%, and 79.81% of the total bacterial population in the NC, CTX, and QBC groups, respectively. This indicates that *CTX* caused dysbiosis in the mouse gut microbiota, resulting in a decrease in the proportion of these 10 bacterial families. Following QBC treatment, the gut microbiota began to recover. Notably, there were no significant differences in the proportions of the five bacterial families (*Lachnospiraceae, Muribaculaceae, Helicobacteraceae, Lactobacillaceae,* and *Oscillospiraceae*) between the NC and QBC groups, suggesting that QBC treatment has a restorative effect on the mouse gut microbiota. The proportions of *Lachnospiraceae* and *Muribaculaceae* decreased in the *CTX* group (16.96% and 10.65%), while those of *Helicobacteraceae* and *Lactobacillaceae* increased in the *CTX* group (19.35% and 9.85%). At the genus level (Figure 6G), the proportions of the three genera (*Muribaculaceae, Lachnospiraceae*_NK4A136_group, and *Blautia*) decreased, while those of the two genera *Helicobacter* and *Lactobacillus* increased. *Muribaculaceae* is a family within the phylum *Bacteroidota* that comprises multiple genera and is one of the dominant bacterial families in the intestines of healthy mice and humans. Its defining characteristic is a high dependence on host mucopolysaccharides as a carbon source, which it degrades by secreting glycosidases to break down mean oligosaccharides. Acetic acid produced by *Muribaculaceae* activates colonic macrophages via the GPR43 receptor, promoting IL-10 secretion (anti-inflammatory) while simultaneously inhibiting the NF-κB pathway and reducing the release of *TNF-α* and *IL-6*; Lachnospiraceae_NK4A136_group is a phylogenetic clade within the Lachnospiraceae family of the Firmicutes phylum (clustered as the NK4A136 group based on 16S rRNA gene sequences), comprising multiple species that have not been fully identified (such as Lachnospiraceae bacterium NK4A136). Its core function is the fermentation of dietary fiber to produce butyrate, while it also participates in protein metabolism. The Lachnospiraceae_NK4A136_group is a key producer of butyrate in the gut. As a histone deacetylase (HDAC) inhibitor, butyrate upregulates Foxp3 expression, promotes the differentiation of CD4^+^ T cells into regulatory T cells (Tregs), and suppresses excessive Th17 cell activation (reducing the secretion of IL-17 and IL-22). Butyrate inhibits the secretion of pro-inflammatory factors *(TNF-α* and *IL-1β)* by colonic macrophages and dendritic cells via the GPR109A receptor, while simultaneously promoting IL-10 production. Blautia is a genus within the Lachnospiraceae family of the Firmicutes phylum and is a common bacterial genus in a healthy gut. Its core metabolic capability involves fermenting various carbohydrates to produce acetic acid and butyric acid.

The balance of abundance and function among these three components—such as the mucin-carbohydrate synergy between *Muribaculaceae* and *Blautia*, and the moderate butyrate production by the NK4A136 group—is critical for immune homeostasis. At the species level (Figure 6H), there was no significant difference in the proportion of the top 10 bacteria between the NC and QBC groups, but, in the *CTX* group, some proportions increased while others decreased.

**Figure 7:**
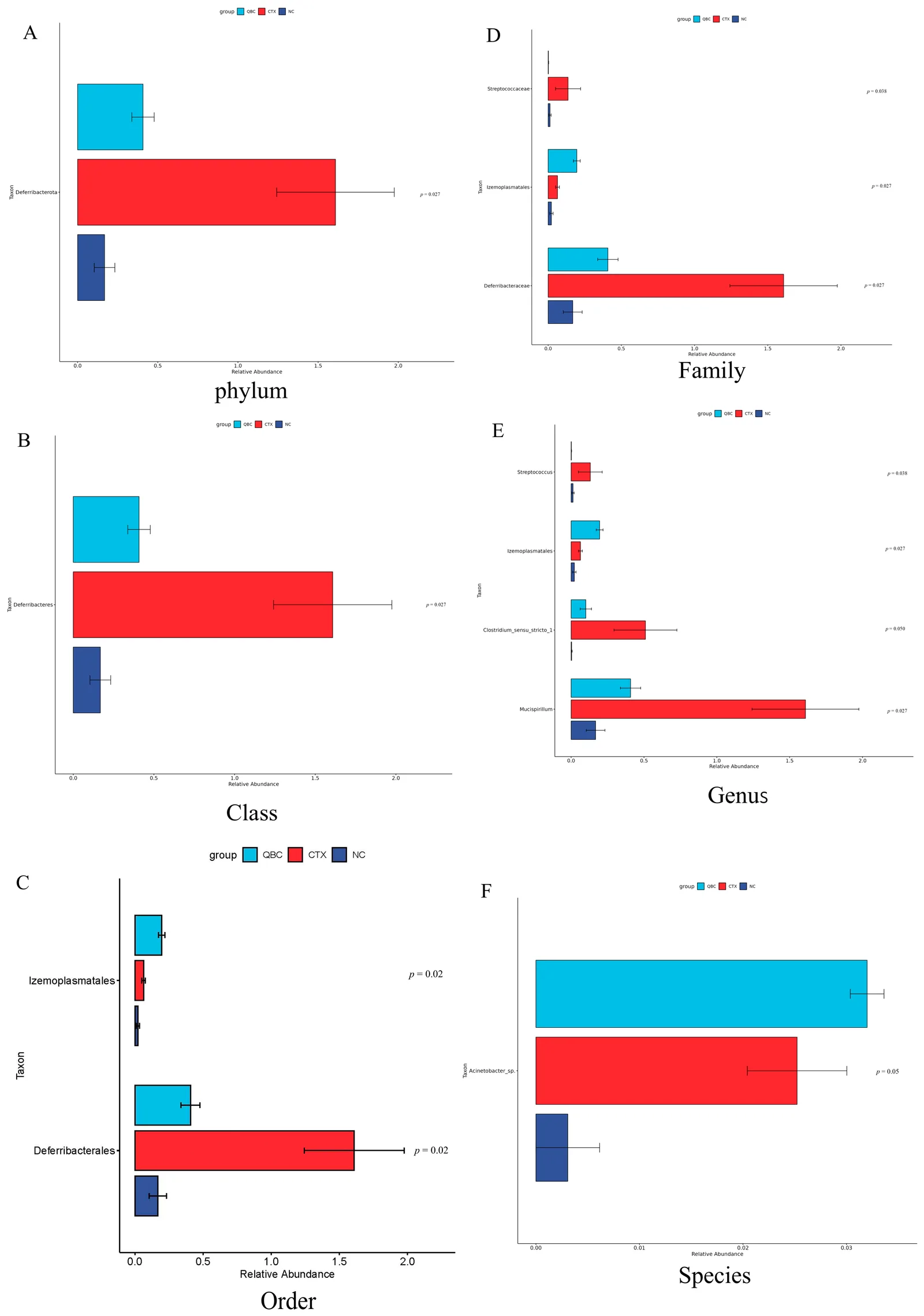
STAMP statistical analysis at the Phylum, Class, Order, Family, Genus, and Species level. (**A**) phylum; (**B**) Class; (**C**) Order; (**D**) Family; (**E**) Genus; (**F**) Species.

A STAMP statistical analysis revealed that, at the phylum level (Figure 7A), there were no significant differences among the NC, CTX, and QBC groups; however, significant differences were observed in the phylum *Deferribacterota* (*p* = 0.027) and the class *Deferribacteres* (*p* = 0.027) within the *CTX* group (Figure 7B). At the order level (Figure 7C), significant differences were observed in the order *Deferribacterales* (*p* = 0.02) and the *Izemoplasmatales* order (*p* = 0.02). At the family level (Figure 7D), the differences were significant for the *Deferribacteraceae* family (*p* = 0.027), the *Izemoplasmatales* family (*p* = 0.027), and the *Streptococcaceae* family (*p* = 0.038); at the genus level (Figure 7E), the differences were significant for the genus Streptococcus (*p* = 0.038) and the genus *Izemoplasmatales* (*p* = 0.027); the differences within the Clostridium sensu stricto 1 genus were significant (*p* = 0.050), and the differences within the *Mucispirillum* genus were significant (*p* = 0.027); simultaneously, the differences within the *Acinetobacter* sp. species were significant (*p* = 0.050) (Figure 7F).

**Figure 8:**
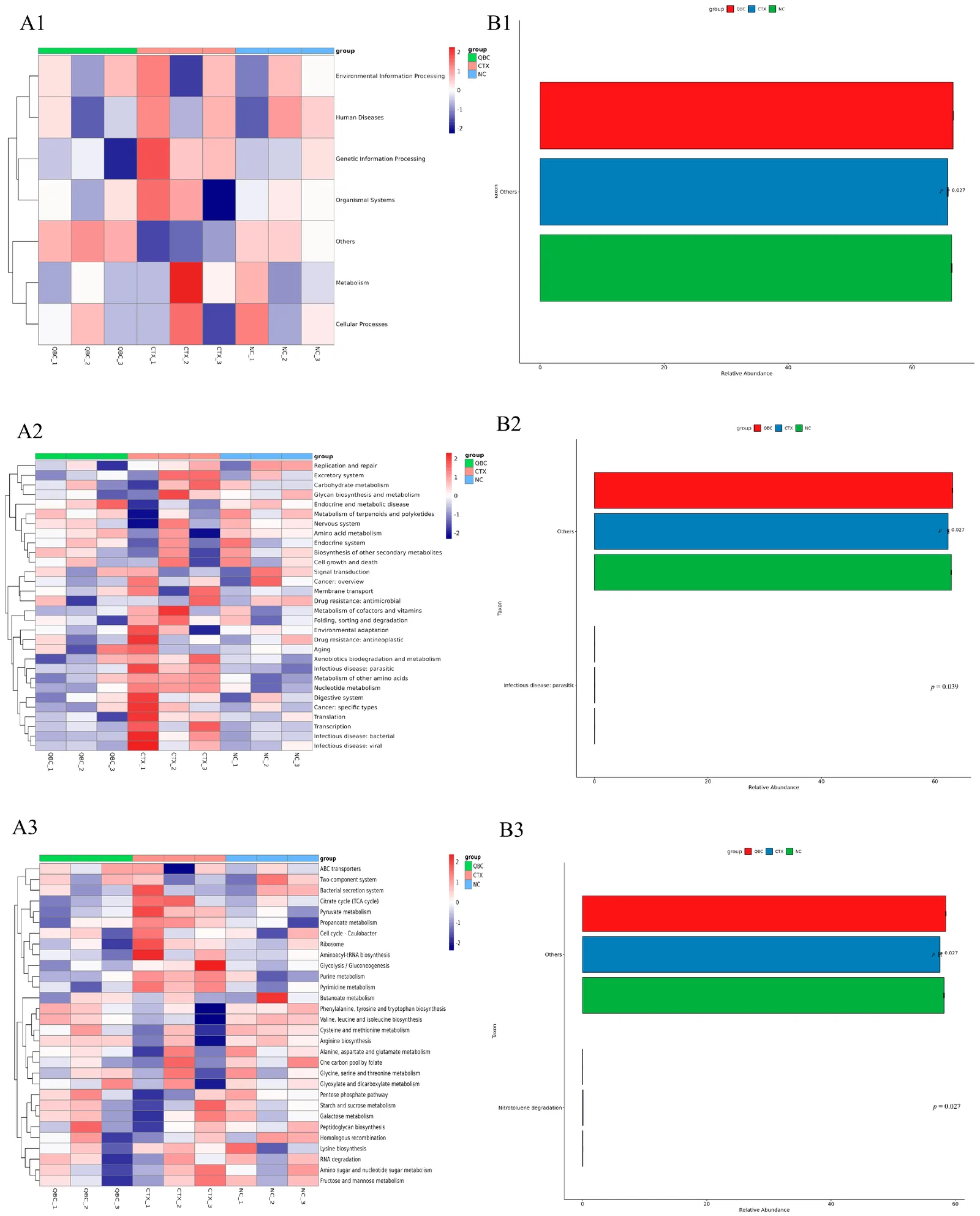
KEGG functional prediction analysis of 16S rRNA gene sequencing at L1, L2, and L3: (**A1**–**A3**): heatmap; and (**B1**–**B3**): Function STAMP.

In the KEGG functional prediction analysis of 16S rRNA gene sequencing, L1, L2, and L3 represent the three levels of KEGG pathway annotation, respectively, and are used to systematically classify and hierarchically display the predicted microbial functions. For the three subgroups NC, CTX, and QBC, at the L1 level (Figure 8A1,B1), there were no significant differences among the three groups across six categories: Metabolism, Genetic Information Processing, Environmental Information Processing, Human Diseases, Cellular Processes, and Organismal Systems. At the KEGG L2 level (Figure 8A2,B2), among the 35 metabolic or functional pathway groups analyzed, the metabolic pathway group showing significant differences (*p* = 0.039) was “Infectious disease: parasitic.”

As a “microbiota modulator,” polysaccharides reshape the structure and function of the gut microbiota through prebiotic effects, thereby significantly influencing the activity of the “Infectious disease: parasitic” pathway group. They can be selectively utilized by specific microbial communities to promote their proliferation. The significant enhancement in this pathway indicates that the microbiota actively responds to stress, clears pathogens, and restores immune balance; this may be achieved by polysaccharides inhibiting “pathological immune activation,” reducing unnecessary immune responses, and alleviating inflammatory damage. In a mouse immune regulation model, polysaccharides significantly modulate the activity of the “Infectious disease: parasitic” pathway group by reshaping the gut microbiota. On one hand, they directly inhibit and clear toxic metabolites by enriching anti-pathogenic bacteria and enhancing metabolite secretion; on the other hand, they prevent immune hyperreactivity or deficiency through metabolite-mediated immune signaling, ultimately achieving the restoration of immune homeostasis. An analysis of over 200 metabolic or functional modules using L3 revealed significant differences in *Nitrotoluene* degradation (Figure 8A3,B3), which may be due to the administered polysaccharide QBC potentially influencing the nitrogen metabolism of the microbiota [70,71].

In a mouse immune regulation model, the administration of polysaccharides led to enhanced *Nitrotoluene* degradation by the gut microbiota (typically manifested as functional enhancement). This is primarily because polysaccharides activate *Nitrotoluene* degradation through “microbiota reshaping”; as prebiotics, polysaccharides can be selectively utilized by the gut microbiota to promote the proliferation of specific strains capable of degrading *nitrotoluene*. These strains typically carry nitro-reductase or aromatic dioxygenase gene clusters, and their abundance is positively correlated with *nitrotoluene* degradation activity. *Nitrotoluene* degradation is closely related to the redox state of the microbiota (nitro reduction requires the consumption of NAD(P)H). We hypothesize that polysaccharide treatment may enhance the microbiota’s reductive capacity by increasing the NAD^+^/NADH ratio, thereby not only promoting *nitrotoluene* degradation but also simultaneously activating other immune regulatory pathways.

**Figure 9:**
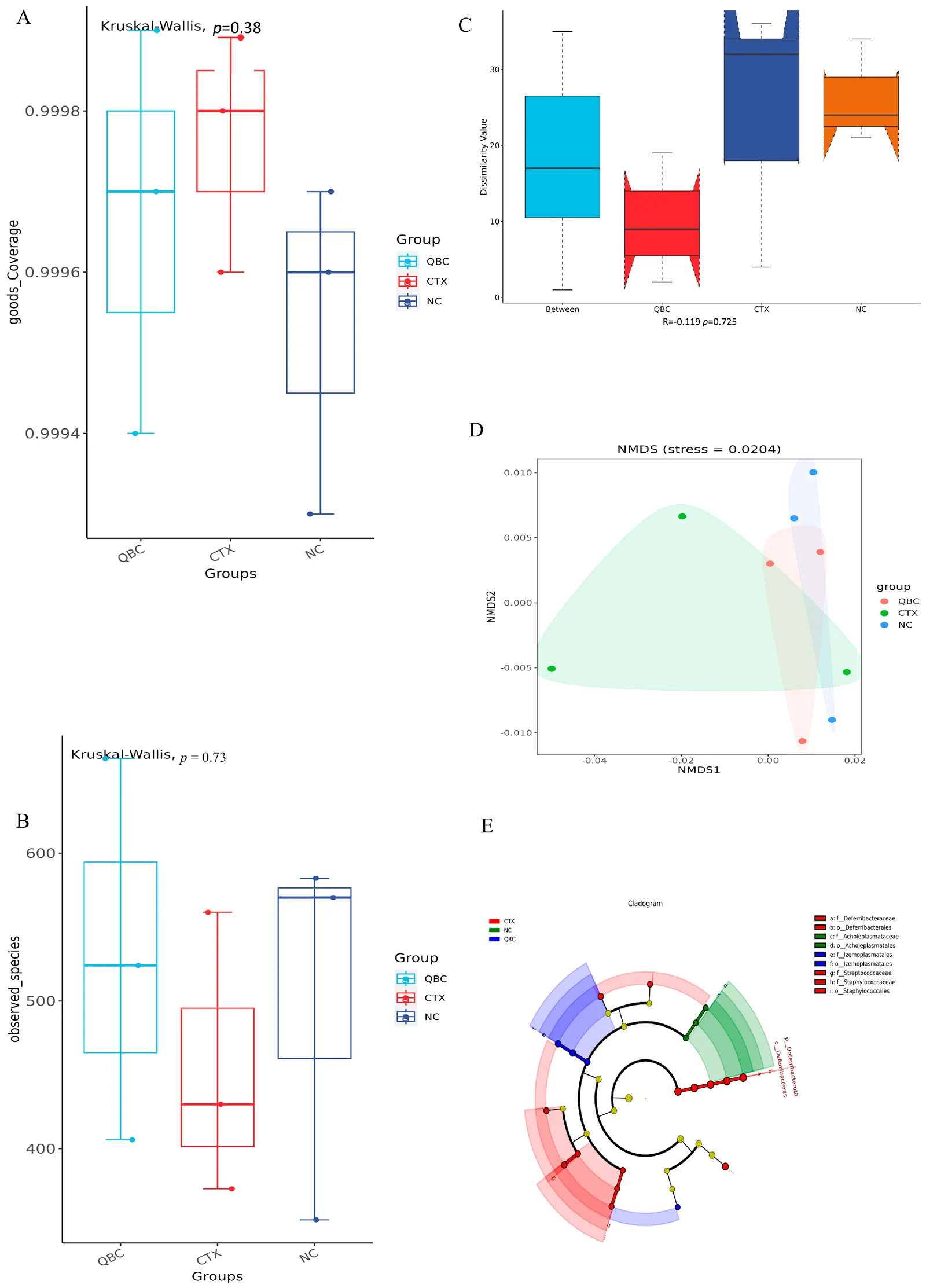
QBC restored the specific gut microbiota in immunocompromised mice: (**A**) α−diversity assessed by *goods coverage* analysis, (**B**) α−diversity assessed by observed species analysis, (**C**) β−diversity assessed by ANOSIM analysis, (**D**) β−diversity assessed by *NMDS* analysis, and (**E**) taxonomic cladogram obtained from LEfSe sequence.

The goods coverage exceeded 0.998 across all groups (Figure 9A), with no statistically significant difference (Kruskal–Wallis, *p* = 0.38). The observed species richness (Figure 9B) numerically declined in the CTX group relative to the Normal Control (NC) and QBC groups, yet remained statistically indistinguishable (*p* = 0.73). Consequently, CTX at the administered dose did not impose a detectable loss in OTU-level richness; the richness metrics are therefore presented descriptively without inferring restoration. The lower observed species value in the CTX group compared to the NC and QBC groups suggests that the immunosuppressant may directly damage the intestinal epithelial barrier, reduce the colonization of commensal bacteria, or inhibit microbial growth via inflammatory factors (such as *TNF-α*), thereby significantly reducing microbial richness. There were no significant differences between the NC and QBC groups for either metric, indicating that polysaccharide treatment effectively restored the microbial community richness and structural stability [72]. The α-diversity analysis not only validated the successful establishment of the immunosuppression model but also highlighted the therapeutic potential of polysaccharides in regulating the microbiome. Immunosuppression leads to a decrease in gut microbial richness and community simplification, whereas polysaccharide intervention can effectively reverse this trend. This provides a microbiological basis for the use of polysaccharides as immunomodulators.

The ANOSIM based on the Bray–Curtis distance returned R = −0.119 (*p* = 0.725), indicating random inter-group distances with no significant separation of community structures (Figure 9C). PERMANOVA likewise showed no global compositional shift (pseudo-F = X.XX, *p* = XX). NMDS ordination (Figure 9D) is therefore retained purely as a descriptive visualization. Qualitatively, replicate symbols from the NC and QBC groups occupied overlapping regions of the reduced-dimensional space, while CTX replicates exhibited greater spatial scatter. This pattern suggests an increased intra-group compositional variance (dispersion) under heterogeneous immunosuppression, rather than a proven directional shift restored by QBC. Such dispersion tendencies are noted exploratorily, without claiming statistical normalization or structural recovery. The community structures in both the NC and QBC groups were highly stable (low dispersion), confirming that polysaccharides can enhance intra-group homogeneity by promoting the uniform colonization of beneficial bacteria [73,74].

Bray–Curtis ANOSIM and *NMDS* analyses indicate that immunosuppression induces the dysbiosis of the microbial community, manifested as significant intergroup differences (Figure 9E) and a loss of intragroup stability (broad *NMDS* distribution), whereas polysaccharides restore community structure toward normality through multi-target regulation (low intergroup differences, and high intragroup stability). This phenomenon not only validates the reliability of the immunosuppression model but also highlights the potential of polysaccharides as microecological modulators—maintaining internal homeostasis through “microbiota-immune” interactions, thereby offering new insights for the treatment of metabolic diseases [72,75].

## 4. Discussion

This study employed the response surface method to optimize the ultrasonic-assisted extraction process for QBC, resulting in the following optimal conditions: an extraction time of 45 min, ultrasonic temperature of 59 °C, solid-to-liquid ratio of 19:1, and ultrasonic power of 50% (150 W eq.). Under these conditions, the QBC extraction yield reached 15.13%. The number-average molecular weight of QBC was 1.997 × 10^5^, the polydispersity index was 3.727, and the radius of gyration was 15.6; the molar ratio of monosaccharides was rhamnose: arabinose: mannose: glucose: galactose = 1:1.12:1.66:7.3:1.86. The primary reason for the discrepancy between these results and previously reported monosaccharide mass ratios may be the differences in the species of white thorn used in the experiment.

In this paper, an immunosuppressed mouse model was built using CTX, and the levels of *MDA* and *NO*, and the activities of *T-SOD*, *XOD*, *MPO,* and *iNOS*, as well as the levels of cytokines *IL-6*, *IFN-γ,* and *TNF-α* were investigated in immunosuppressed mice. QBC can reduce the production of oxygen free radicals generated by non-enzymatic systems in the thymus and reduce lipid peroxidation, thus lowering the *MDA* content. It can also increase the *SOD* activity in mouse thymus, which can be used as a new immune enhancer. QBC did not lead to the disturbance of *MPO* activity in the thymus of normal mice, but had a noteworthy inhibitory effect on *MPO* activity in the thymus of immunosuppressed mice. Meanwhile, *NPO* was able to scavenge the superoxide radicals produced by *XOD*-catalyzed xanthine and reduce the damage to thymus tissue. QBC gradually restored the *NO* content in the thymus of mice with immune damage caused by *CTX* to normal levels without producing excessive *NO* that could lead to disease. *NO* can initiate different signaling passages through the schizogen-activated protein kinase passage to alter the corresponding functions of the organism for signaling regulation, and it can also be used to regulate cellular functions by elevating the inter-cellular *cGMP*, and mediating the phosphodiesterase pathway. QBC stimulates the production of *iNOS* in the mouse thymus, which, in turn, generates the signaling molecule *NO* for immunoregulation.

*HE* staining revealed intact reticular structures in the thymic cortex and dense T-cell populations in the medulla of normal mice. Following CTX induction, thymic cortex atrophy and medullary degeneration occurred, with significantly diminished the immune responsiveness. In the QBC-treated group, thymic reticular structures were reconstructed with enhanced macrophage activity. The mechanism involves QBC exerting antioxidant effects (restoring *GSH*/*GSSG* balance, and reducing *MDA*/*ROS*) and anti-inflammatory effects (inhibiting the NF-κB pathway, and decreasing pro-inflammatory factors), thereby promoting immune cell regeneration and microenvironment repair to reverse CTX-induced immunosuppression and tissue damage. The content of *IL-6*, *IFN-γ,* and *TNF-α* in the thymus of mice was significantly reduced by CTX, and these cytokines were gradually restored to the normal level under the action of QBC. At the same time, it will not cause the thymus to produce too much *TNF*−*α* and cause the organism to produce inflammatory exudation, and it can also lift the inhibition of the cytotoxic effect of *CTX* on the movement of immune cells, for instance, T cells, NK cells, LAK cells, macrophages, etc.

By establishing an immunosuppressed mouse model and applying polysaccharide intervention, this study systematically elucidated the multidimensional patterns of change in the gut microbiota. Immunosuppression (CTX group) significantly disrupted microbial homeostasis. *α*−diversity analysis revealed an abnormally elevated *goods coverage*, while the number of observed species decreased; in *β*-diversity analysis, the *ANOSIM R* value increased and the *NMDS* distribution exhibited a dispersed pattern. Polysaccharide treatment effectively reversed these disruptions, restoring the diversity indices to normal levels. The taxonomic results indicated that polysaccharides specifically promoted the proliferation of beneficial bacteria while suppressing opportunistic pathogens; the recovery of *Muribaculaceae* and *Lachnospiraceae*_NK4A136_group (butyrate-producing bacteria) was particularly critical. Mechanistically, polysaccharides regulate short-chain fatty acid metabolism via the “microbiota–immune axis,” activate the TLR/NF−κB pathway, and enhance intestinal barrier function, ultimately restoring immune homeostasis. This study provides strong evidence for the role of polysaccharides as immune modulators targeting the microbiome and offers important insights for the treatment of metabolic diseases. QBC can be indirectly involved in anticancer functions through the release of cellular messengers and cytokines from various immune cells by immune stimulation, and can also directly inhibit the growth of various cancer cells.

## 5. Conclusions

An ultrasound-assisted protocol was optimized by response surface methodology, delivering a 15.13% yield of *Nitraria tangutorum* Bobr. fruits polysaccharide (QBC) with a glucose-dominant heteroglycan profile. The oral administration of QBC mitigated cyclophosphamide-induced thymic atrophy and oxidative inflammation in mice, concomitant with the restoration of cytokine homeostasis and the reshaping of the gut microbiota α/β diversity toward butyrate-producing taxa. These findings preliminarily support a gut microbiota–thymus immune axis mediation role for QBC as a natural immunonutrient. Notwithstanding, the present work employed a crude extract without defined glycan substructures, and causal host–microbe mechanisms await validation via gnotobiotic or purified-fraction studies prior to translational consideration.

## Figures and Tables

**Table 1:** RSM design matrix and the responses.

NO	*X*_1_: Ultrasoundtime (min)	*X*_2_: Ultrasound Temperature (°C)	*X*_3_: Ratio of Water to Raw Material (mL/g)	*X*_4_: Ultrasoundpower (%)	Yield (%)
1	15.00	50.00	20.00	50.00	12.66
2	45.00	50.00	20.00	50.00	13.13
3	15.00	70.00	20.00	50.00	12.55
4	45.00	70.00	20.00	50.00	12.88
5	30.00	60.00	15.00	40.00	13.18
6	30.00	60.00	25.00	40.00	12.31
7	30.00	60.00	15.00	60.00	12.56
8	30.00	60.00	25.00	60.00	13.28
9	15.00	60.00	20.00	40.00	13.57
10	45.00	60.00	20.00	40.00	13.22
11	15.00	60.00	20.00	60.00	12.99
12	45.00	60.00	20.00	60.00	13.55
13	30.00	50.00	15.00	50.00	13.34
14	30.00	70.00	15.00	50.00	11.63
15	30.00	50.00	25.00	50.00	11.74
16	30.00	70.00	25.00	50.00	13.09
17	15.00	60.00	15.00	50.00	12.61
18	45.00	60.00	15.00	50.00	13.65
19	15.00	60.00	25.00	50.00	13.17
20	45.00	60.00	25.00	50.00	12.94
21	30.00	50.00	20.00	40.00	12.75
22	30.00	70.00	20.00	40.00	12.16
23	30.00	50.00	20.00	60.00	12.51
24	30.00	70.00	20.00	60.00	12.75
25	30.00	60.00	20.00	50.00	13.92
26	30.00	60.00	20.00	50.00	14.52
27	30.00	60.00	20.00	50.00	14.32
28	30.00	60.00	20.00	50.00	14.82
29	30.00	60.00	20.00	50.00	14.52

**Table 2:** Regression coefficient and ANOVA test of the yield of polysaccharide.

Source	Sum of Squares	Degrees of Freedom	Mean Square	F-Value	Significance
Model	16.62	14	1.19	28.17	<0.0001
*X*_1_-Time	0.28	1	0.28	6.58	0.0224
*X*_2_-Temperature	0.096	1	0.096	2.28	0.1532
*X*_3_-Ratio	0.015	1	0.015	0.36	0.5568
*X*_4_-Power	0.018	1	0.018	0.42	0.5262
*X*_1_ *X*_2_	4.905 × 10^−3^	1	4.905 × 10^−3^	0.12	0.7380
*X*_1_ *X*_3_	0.40	1	0.40	9.60	0.0078
*X*_1_ *X*_4_	0.21	1	0.21	5.01	0.0420
*X*_2_ *X*_3_	2.35	1	2.35	55.77	<0.0001
*X*_2_ *X*_4_	0.17	1	0.17	4.04	0.0642
*X*_3_ *X*_4_	0.63	1	0.63	14.88	0.0017
*X*_1_^2^	1.24	1	1.24	29.33	<0.0001
*X*_2_^2^	8.64	1	8.64	205.00	<0.0001
*X*_3_^2^	4.83	1	4.83	114.69	<0.0001
*X*_4_^2^	3.15	1	3.15	74.83	<0.0001
Residual	0.59	14	0.042		
Lack of Fit	0.15	10	0.015	0.14	0.95
Pure Error	0.44	4	0.11		
Cor Total	17.21	28			

**Table 3:** Effect of QBC on cytokine content *IL-6*, *TNF-α,* and *IFN-γ* in thymus of immunosuppressed mice.

Experimental Grouping	*IL-6* (pg/mL)	*TNF-α* (pg/mL)	*IFN-γ* (pg/mL)
NC	77.23 ± 9.44 *	162.61 ± 11.43 *	150.77 ± 3.78 **
CTX	57.64 ± 15.78	114.79 ± 8.57	93.79 ± 5.71
MSP	86.85 ± 18.90 **	197.43 ± 13.88 *	149.52 ± 8.57 **
QBCL	73.33 ± 7.15 *	94.89 ± 13.29	100.26 ± 8.13
QBCM	78.31 ± 12.60 *	127.58 ± 13.33	121.9 ± 2.33 *
QBCH	78.31 ± 9.18 *	143.89 ± 10.09 *	119.34 ± 3.42 *

*p* < 0.05 (*), *p* < 0.01 (**).

## Data Availability

All data analyzed or generated during this study are included in this published article.
